# Micro-CT Evaluation of Marginal and Internal Fit of Lithium Disilicate Crowns – Influence of Wax-up and Manufacturing Technique: Marginal and Internal Fit of Lithium Disilicate Crowns

**DOI:** 10.31661/gmj.v13iSP1.3562

**Published:** 2024-12-08

**Authors:** Soheil Hariri, Ali Jamali Ghomi, Seyed Mohammad Reza Hakimaneh, Mohammad Amin Bafandeh, Maryam Jahangiri, Sayed Shojaedin Shayegh

**Affiliations:** ^1^ Department of Prosthodontics, Faculty of Dentistry, Zanjan University, Zanjan, Iran; ^2^ Department of Prosthodontics, Faculty of Dentistry, Shahed University, Tehran, Iran

**Keywords:** Lithium Disilicate, Crown, Milling, 3Dprint

## Abstract

**Background:**

Lithium disilicate crowns are widely used in dentistry, with various fabrication methods available. However, there is a research gap in comparing the marginal and internal fit of these crowns produced through different manufacturing techniques.This study aims to evaluate the impact of various manufacturing methods on the marginal and internal fit of lithium disilicate crowns.

**Materials and Methods:**

The left maxillary canine’s acrylic tooth was prepared using a high-speed handpiece. Subsequently, the prepared tooth was scanned with a laboratory scanner, and 40 dies were milled with resin. These dies were then divided into four groups (n=10), with lithium disilicate crowns fabricated using different methods for each group: group 1) conventional wax-up method and heat press, group 2) 3D print wax-up method and heat press, group 3) wax-up milling method and heat press, and group 4) CAD/CAM method. The marginal and internal fit of the crowns were assessed using micro-CT by measuring Absolute Marginal Discrepancy (AMD), Marginal Gap (MG), Axial Gap (AG), and Occlusal Gap (OG) at various points. Group comparisons were conducted using one-way ANOVA, while Spearman rank correlation coefficient was used to evaluate variable correlations (α=0.05).

**Results:**

ANOVA analysis indicated significant differences among all groups for most examined points except for lingual AMD. In inter-group comparisons, the CAD/CAM method demonstrated superior results in MG buccal, MG lingual, AMD buccal, AG2, AG3, OG1, OG3, and OG4 measurements. The 3D printing method outperformed in AG1 and OG2 comparisons while the milling method excelled in AG4 comparison. Although no significant difference was observed in lingual AMD comparison among groups, the CAD/CAM approach exhibited better average agreement. Overall, the conventional group displayed the weakest performance in terms of adaptation.

**Conclusion:**

The study findings suggest that all-digital and semi-digital methods for fabricating lithium disilicate crowns offer better adaptation compared to conventional techniques. Among the evaluated methods, the conventional approach showed the lowest level of adaptation overall.

## Introduction

In contemporary dentistry, all-ceramic single crowns have demonstrated comparable survival rates to metal-ceramic crowns over a five-year period [1]. However, metal-ceramic restorations present drawbacks such as galvanic destruction, corrosion, gum discoloration, and periodontal issues [2][3]. These restorations cannot satisfy all patients due to the lack of high-level aesthetic needs and the time-consuming preparation process [4]. These limitations have led to a preference for metal-free alternatives like Lithium disilicate glass-ceramic (LDC) due to their superior aesthetic appeal and simplified preparation process [5].

All-ceramic restorations can be categorized into glass-based ceramics (e.g., feldspathic porcelain, leucite, LDC), glass-infiltration ceramics, and non-glass-based ceramics (e.g., Alumina and Zirconia). Among these, glass-based ceramics are popular for their biocompatibility, marginal adaptation, polishing properties, and aesthetic advantages [6]. Lithium disilicate glass-ceramic is a prominent choice within this category and has evolved into two generations represented by IPS Empress 2 (first generation) and IPS e.max press (second generation) [7]. The second-generation material exhibits enhanced mechanical and aesthetic characteristics such as strength, elasticity modulus, color range, surface texture, marginal fit precision, and translucency [6][7][8]. Notably, the introduction of the e.Max line in 2009 led to the discontinuation of Empress 2 production [9]. IPS e.max press crowns are fabricated using the lost wax technique involving wax pattern creation followed by mold formation in refractory investment material [10][11].

The core of IPS e.max comprises a lithium disilicate framework with a glass-ceramic fluorapatite layered structure [12]. This core undergoes pressing at 920 degrees Celsius before being layered with a glass matrix containing diffused apatite crystals [12][13][14][15]. Compared to earlier generations of glass ceramics, IPS e.max press demonstrates significantly higher strength while maintaining aesthetic appeal. Guess et al [16] indicated that IPS emax press has extraordinary durability and a bending strength of 360 MPa. The success of dental restorations hinges on factors like esthetics, fracture resistance, and marginal integration. Marginal adaptation plays a crucial role in ensuring the longevity of both the abutment tooth and the restoration itself [17].

Also, McLean suggested a limit of 120 microns for acceptable marginal ill-fit [18] and recent studies approved this limitation [19][20][21]. Achieving minimal marginal gaps is essential for clinical success in dental treatments as improper adaptation can lead to issues like cement washout, plaque accumulation, microleakage, and secondary caries [22][23]. Maintaining proper marginal fit is vital for preventing complications like gingivitis related to restoration defects such as margin roughness or inadequate adaptation [24].

In the conventional methods of fabricating lithium disilicate restorations, two primary techniques are commonly employed: the heat press-lost wax technique (IPS e.max Press) and the CAD/CAM technique (IPS e.max CAD) [25]. In IPS e.max Press method, first, the restoration wax pattern is made. After that, it is placed in the furnace to remove the wax. After removing the wax, the desired ingots are injected using centrifugal force [26]. IPS e.max Press method has some disadvantages include thermal sensitivity, dimensional change, elastic memory, and high coefficient of thermal expansion and skill sensitivity. New techniques for wax modeling can help overcome these limitations [27].

On the other hand, CAD/CAM systems offer numerous advantages including enhanced restoration quality, improved accuracy by Despite its benefits, CAD/CAM systems have limitations that may impact marginal adaptation due to factors like scanner camera quality, software restoration design constraints, and milling limitations. Complex features like feather-edge finish lines or intricate occlusal surfaces may not be suitable for milling. Additionally, improper internal compatibility can result from the size of the milling cutter [28][29].

In various studies, the amount of marginal discrepancy is lower than internal discrepancy [24].Another emerging method involves creating 3D-printed wax patterns by layering and solidifying liquid resin through radiation exposure. This approach offers advantages such as cost-effectiveness compared to reduction methods, minimal material wastage during production, capability to produce intricate internal geometries and fine details including undercuts, and simultaneous printing of multiple materials [30][31].

The null hypothesis is no differences between the marginal and internal compatibility of lithium disilicate crowns with various manufacturing methods. This study aims to compare the marginal and internal compatibility of lithium disilicate crowns fabricated using conventional wax-up with heat press technique against 3D print wax-up with heat press technique, wax-up milling with heat press technique, and CAD/CAM method.

## Materials and Methods

**Figure-1:**
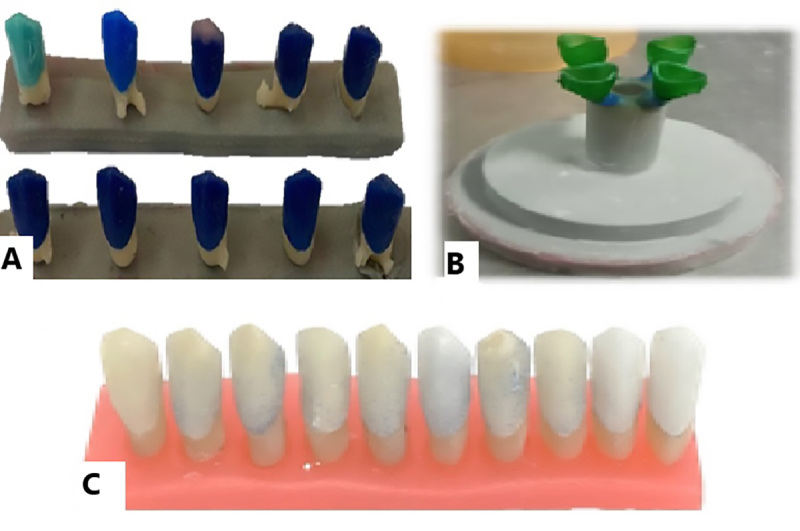
Figure 1. Conventional method. A,10 wax patterns made by conventional method on 10 resin dies. B, Sprue forming of the wax patterns. C, 10 pieces of lithium disilicate crowns (IPS Emax press) obtained by the conventional waxing method on 10 pieces of resin dies.

**Figure-2:**
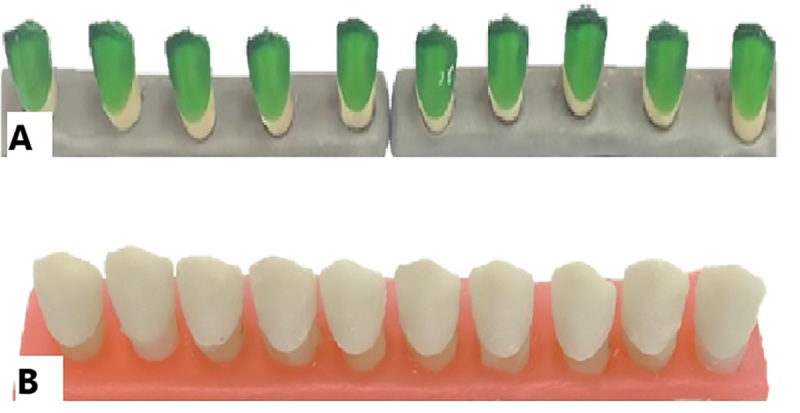
Figure 2. Digital wax-up (3D Printer) and conventional casting. A, 10 digital resin patterns by 3D printing method on 10 resin dies. B, 10 pieces of lithium disilicate crowns (IPS Emax press) obtained by 3D printing method of resin pattern on 10 pieces of resin dies.

**Figure-3:**
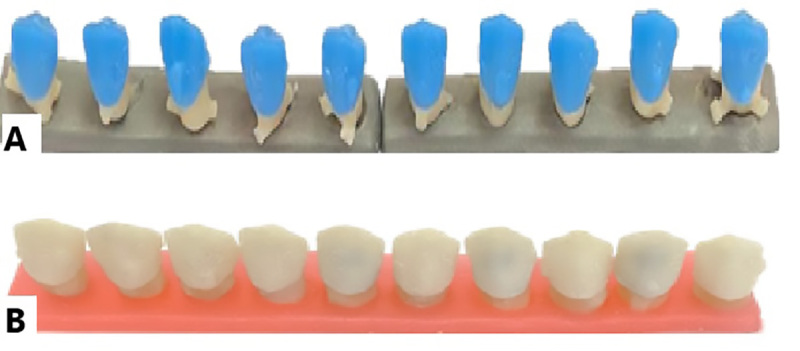
Figure 3. Digital wax-up (Milling) and conventional casting. A, 10 digital wax patterns by print-milling method on 10 resin dies. B, 10 pieces of lithium disilicate coating (IPS Emax press) obtained by milling the wax pattern on 10 pieces of resin dies.

**Figure-4:**
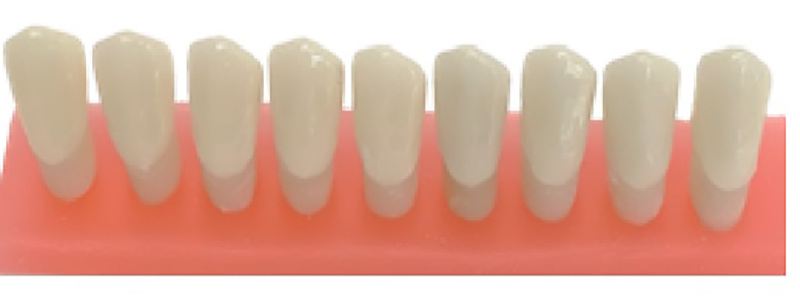
Figure 4. Fully Digital (Milling). 10 pieces of lithium disilicate crowns (IPS Emax CAD) obtained by CAD/CAM method on 10 pieces of resin dies

**Figure-5:**
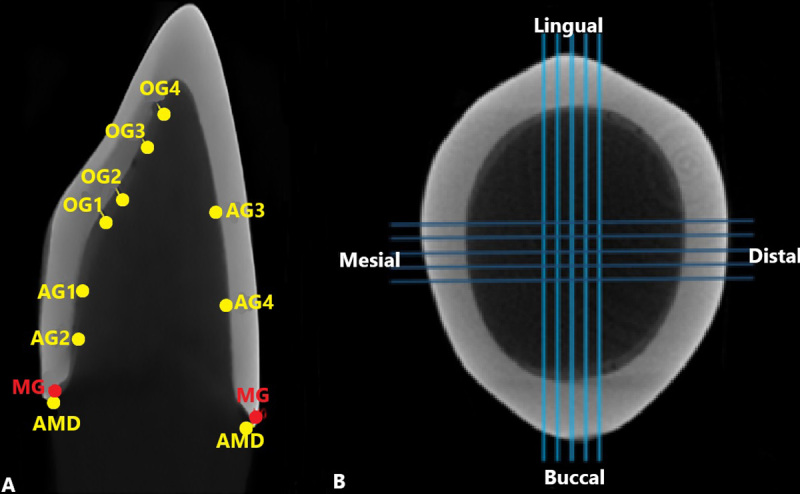
Figure 5. A, Marginal gap(MG) and absolute marginal discrepancy(AMD) differences measurement. B, Measurement of occlusal and axial gap in vertical line from ceramic crown to master die.

**Table:** Table1. Measured Points in Each Crown Vertical Cut for Internal and Marginal Matching Micro-CT Evaluation

**Measured Points**		**CAD-CAM**	**Milling**	**3DPrint**	**Conventional**	**Total**
**Absolute Marginal Discrepancy (AMD)**	AMD buccal	10	10	10	10	40
	AMD lingual	10	10	10	10	40
**Marginal Gap (MG)**	MG buccal	10	10	10	10	40
	MG lingual	10	10	10	10	40
	OG1	10	10	10	10	40
**Occlusal Gap (OG)**	OG2	10	10	10	10	40
	OG3	10	10	10	10	40
	OG4	10	10	10	10	40
	AG1	10	10	10	10	40
**Axial Gap (AG)**	AG2	10	10	10	10	40
	AG3	10	10	10	10	40
	AG4	10	10	10	10	40
AMD+MG+OG+AG	**Total **	120	120	120	120	480

In this original research, the educational model (dental study model 500A; Nissin) was used. The canine acrylic tooth on the left side of the maxilla was selected for the preparation of a single-unit fixed prosthesis. To have a digital index, the model was scanned using a SMART laboratory scanner (Open Technology), and the impression model used polyvinyl siloxane (Panasil) before tooth preparation. Having digital and conventional index were essential for digital and manual wax up methods. The acrylic tooth was prepared using a high-speed handpiece with water spray and a rough Tapered round-end diamond bur for initial preparation, followed by a softer bur to refine the preparation surfaces. The depth of axial and occlusal reduction was 1.5 mm and 2 mm, respectively, and a chamfer finishing line was created 360 degrees [32]. Then, 40 prepared teeth PMMA dies (TopZir, Biotech Co.) were milled using a milling machine (Versamill 5X200; Axsys Dental Solutions). The dies were randomly divided into 4 groups of 10.

### First Group. Conventional Method

polyvinyl siloxane (PVS) impressions were taken using plastic trays and a two-step putty wash method. Impressions were poured with type IV stone plaster (Fujirock; GC), trimmed, and grooved. Die spacer (TruFit, George Taub Products) was applied for cement space 4 times. Dies were prepared with a dipping and waxing method (Green, Bego). Wax patterns were molded, cast, and sprued for IPS Empress cylinders. A specialized ring IPS e.max Investment Ring System) was used for cylindering at high temperatures to create restorations matching the die. The cylinder was heated up to 800 degrees Celsius to evaporate and remove the wax pattern. The ceramic ingot was inserted into the sprue, and after heating to a temperature of 920 degrees Celsius, the molten ceramic was pressed and vacuumed inside the mold. After pressing, the surface of the restoration was cut with abrasive particles and sprue, and then the crown was matched to the die (Figure-1).

### Second Group. Digital Wax-up (3D Printer) and Conventional Casting

Polyvinyl siloxane (PVS) impressions were taken with plastic molding trays and a two-step putty wash impression method. Impressions were poured with type IV stone plaster (Fujirock; GC) using a vacuum mixer (Multivac 4, Degussa) as per manufacturer recommendations. In Exocad design software (Exocad, Align Technology, Darmstadt, Germany. Crowns were 3D printed using wax (Press E CastR; EnvisionTEC Inc) and conventionally cast following procedures similar to the first group (Figure-2).

### Third Group. Digital Wax-up (Milling) and Conventional Casting

In this group, a combination of digital wax-up (milling) and conventional casting techniques were utilized. The process involved importing scanned images into the Exocad design software (Exocad, Align Technology, Darmstadt, Germany (and determining the cement space with a thickness of 30 microns. To create the crowns, a full anatomical design approach was adopted, utilizing the initial scan taken prior to tooth preparation. The designed crowns were then milled using the Versamill 5X200 milling method (Axsys Dental Solutions) and wax disks (Zirkohnzahn Wax Purple 98H16, ZirconZahn; An der Ahr). For the conventional casting process, the same approach as the first group was followed (Figure-3).

### The Fourth Group. Fully Digital (Milling)Impression Method

In this group, a fully digital approach utilizing the milling technique was employed. All molds were evaluated by the same operator to ensure consistency and accuracy. To create the PVS casts, type IV gypsum (Fujirock; GC) was used and mixed with water according to the manufacturer’s instructions. A vacuum mixer (Multivac 4, Degussa) was utilized to ensure proper mixing. Within the Exocad design software (Exocad, Align Technology), the cement space was determined with a thickness of 30 microns. The crowns were designed to be fully anatomical, based on the initial scan performed before preparing the original model. For the crown design, the milling method was employed using the Versamill 5X200 (Axsys Dental Solutions) machine. Lithium disilicate disks (IPS e.max CAD A2, Ivoclar Vivadent) were used in the milling process (Figure-4).

### Method of Internal and Marginal Adaptation Evaluation

To assess the internal and marginal adaptation of the crowns fabricated using the aforementioned methods, a thorough evaluation and testing process was conducted.

A reference sample (dental study model 500A; Nissin) was used to ensure complete seating of the crowns before measurement. The evaluation was carried out using the micro-computed tomography (micro-CT) method, which utilizes X-rays to measure the internal and marginal fit of the crowns.

This method offers several advantages, including high-resolution images and the ability to measure in three dimensions.To maintain consistency, the main model for micro-CT was securely fixed on a screen, ensuring the same positioning for all samples.

The restorations, without the use of cement, were then carefully and without force placed on the original model. Subsequently, a micro-CT scan was performed using specific parameters, including 70 kVp, 114 µA, and an integration time of 300 ms. The scanned images were processed in software to evaluate the marginal and internal fit of the crowns.

Following the methodology described in the study by Mously et al. [33], twelve points were examined in each vertical cut.

These points included two points related to the marginal gap, two points related to absolute marginal discrepancy, four points related to occlusal distance, and four points related to axial distance. In total, 120 points were measured for each group, providing a comprehensive analysis of the adaptation (Table-1, Figure-5).

## Results

**Table:** Table2. Analysis of Variance (ANOVA) of the Matching of Crowns in Four Groups: 3D-print, CAD/CAM, Conventional and Milling in the Buccolingual View

**ANOVA**
		Sum of Squares	df	Mean Square	F	Sig.
MG buccal	Between Groups	157736.373	3	52578.791	244.422	.000
	Within Groups	7744.134	36	215.115		
	Total	165480.506	39			
MG lingual	Between Groups	180323.944	3	60107.981	337.068	.000
	Within Groups	6419.743	36	178.326		
	Total	186743.687	39			
AMD buccal	Between Groups	116193.863	3	38731.288	171.457	.000
	Within Groups	8132.213	36	225.895		
	Total	124326.077	39			
AMD lingual	Between Groups	26666140520000.000	3	8888713507000.000	1.000	.404
	Within Groups	319942905500000.000	36	8887302929000.000		
	Total	346609046000000.000	39			
AG1	Between Groups	114692.467	3	38230.822	66.548	.000
	Within Groups	20681.339	36	574.482		
	Total	135373.806	39			
AG2	Between Groups	91570.318	3	30523.439	58.260	.000
	Within Groups	18861.088	36	523.919		
	Total	110431.406	39			
AG3	Between Groups	94479.050	3	31493.017	82.785	.000
	Within Groups	13695.139	36	380.421		
	Total	108174.189	39			
AG4	Between Groups	117503.795	3	39167.932	148.095	.000
	Within Groups	9521.194	36	264.478		
	Total	127024.989	39			
OG1	Between Groups	133564.169	3	44521.390	120.618	.000
	Within Groups	13287.949	36	369.110		
	Total	146852.117	39			
OG2	Between Groups	178419.225	3	59473.075	149.394	.000
	Within Groups	14331.461	36	398.096		
	Total	192750.687	39			
OG3	Between Groups	150558.248	3	50186.083	192.822	.000
	Within Groups	9369.755	36	260.271		
	Total	159928.004	39			
OG4	Between Groups	150813.532	3	50271.177	219.713	.000
	Within Groups	8236.938	36	228.804		
	Total	159050.469	39			

**Table:** Table3. Mean and Standard Deviation of the Degree of Matching of Crowns in Four Groups: 3D-print, CAD/CAM, Conventional and Milling in the Buccolingual View

**Descriptive**
		N	Mean	Std. Deviation	Std. Error Lower Bound	95% Confidence Interval for Mean		Minimum	Maximum
						Upper Bound			
MG buccal	3DPrint	10	3.7489	1.54052	.48715	2.6469	4.8509	2.64	6.88
	cadcam	10	3.4266	1.05950	.33504	2.6686	4.1845	1.79	5.44
	conventional	10	149.4274	29.18888	9.23033	128.5469	170.3079	101.45	197.65
	milling	10	6.0980	2.22996	.70518	4.5027	7.6932	3.06	11.18
	Total	40	40.6752	65.13900	10.29938	19.8427	61.5077	1.79	197.65
MG lingual	3DPrint	10	3.8747	2.20414	.69701	2.2980	5.4515	1.01	7.81
	cadcam	10	3.8504	1.27286	.40251	2.9399	4.7610	2.47	6.82
	Conventional	10	159.3112	26.39944	8.34823	140.4262	178.1962	118.61	197.61
	milling	10	5.0444	3.14582	.99479	2.7940	7.2947	1.69	12.36
	Total	40	43.0202	69.19754	10.94109	20.8897	65.1506	1.01	197.61
AMD buccal	3DPrint buccal	10	5.0774	2.25539	.71322	3.4640	6.6909	2.22	9.46
	cadcam buccal	10	4.7906	1.92730	.60947	3.4119	6.1693	1.11	7.40
	conventional buccal	10	130.3715	29.79153	9.42091	109.0600	151.6831	85.65	176.34
	milling buccal	10	7.9370	2.69118	.85103	6.0119	9.8622	4.01	12.22
	Total	40	37.0442	56.46103	8.92727	18.9870	55.1013	1.11	176.34
AMD lingual	3DPrint buccal	10	5.7229	3.58178	1.13266	3.1607	8.2852	2.00	14.98
	cad cam buccal	10	4.5699	1.94791	.61598	3.1765	5.9634	1.58	7.67
	conventional buccal	10	188.2670	115.96900	188.85900	178.6370	189.1710	100.92	188.54654
	milling buccal	10	7.0651	3.20200	1.01256	4.7746	9.3557	2.97	13.72
	Total	40	471405.6562	2981176.18300	471365.34230	-482020.7416	1424832.0540	1.58	18854654.00
AG1	3DPrint buccal	10	3.2099	2.87384	.90879	1.1541	5.2658	1.04	9.54
	cad cam buccal	10	3.3928	1.86596	.59007	2.0580	4.7277	1.08	7.11
	conventional buccal	10	127.0279	47.78961	15.11240	92.8413	161.2145	60.13	220.55
	milling buccal	10	3.4951	1.52930	.48361	2.4011	4.5891	1.53	6.56
	Total	40	34.2814	58.91624	9.31548	15.4391	53.1238	1.04	220.55
AG2	3DPrint buccal	10	2.5657	2.18671	.69150	1.0014	4.1300	1.24	8.68
	cad cam buccal	10	2.5060	1.36502	.43166	1.5295	3.4825	1.01	5.08
	conventional buccal	10	113.1998	45.69518	14.45008	80.5114	145.8881	60.42	188.64
	milling buccal	10	3.0426	.99095	.31337	2.3337	3.7515	1.34	4.76
	Total	40	30.3285	53.21254	8.41364	13.3103	47.3467	1.01	188.64
AG3	3DPrint buccal	10	4.0849	1.46401	.46296	3.0376	5.1322	2.58	7.67
	cad cam buccal	10	3.3730	2.22187	.70262	1.7835	4.9624	1.25	6.78
	conventional buccal	10	115.8862	38.90411	12.30256	88.0559	143.7166	74.80	193.45
	milling buccal	10	3.4942	1.03561	.32749	2.7533	4.2350	1.93	5.32
	Total	40	31.7096	52.66590	8.32721	14.8662	48.5529	1.25	193.45
AG4	3DPrint buccal	10	3.4890	1.87961	.59438	2.1444	4.8336	1.50	8.06
	cad cam buccal	10	2.8898	1.85680	.58717	1.5615	4.2180	.93	7.49
	conventional buccal	10	128.2549	32.40961	10.24882	105.0705	151.4394	88.65	183.36
	milling buccal	10	2.8842	.73959	.23388	2.3551	3.4132	1.47	3.77
	Total	40	34.3795	57.07058	9.02365	16.1274	52.6315	.93	183.36
OG1	3DPrint buccal	10	7.8597	2.22525	.70369	6.2679	9.4516	4.09	11.30
	cad cam buccal	10	7.3112	4.35882	1.37838	4.1931	10.4293	2.56	14.85
	conventional buccal	10	142.7790	37.91183	11.98877	115.6585	169.8995	76.70	219.78
	milling buccal	10	13.1295	3.89631	1.23212	10.3422	15.9167	5.18	18.49
	Total	40	42.7699	61.36317	9.70237	23.1450	62.3948	2.56	219.78
OG2	3DPrint buccal	10	6.9844	2.27336	.71890	5.3582	8.6107	4.09	10.64
	cad cam buccal	10	7.8871	4.31924	1.36586	4.7973	10.9769	2.56	15.17
	conventional buccal	10	163.8358	39.39814	12.45879	135.6520	192.0195	125.64	256.85
	milling buccal	10	14.3406	4.04312	1.27855	11.4483	17.2328	7.19	20.06
	Total	40	48.2620	70.30167	11.11567	25.7784	70.7455	2.56	256.85
OG3	3DPrint buccal	10	7.3527	2.11776	.66969	5.8377	8.8677	4.23	10.70
	cad cam buccal	10	6.4093	3.45568	1.09278	3.9372	8.8813	1.52	12.73
	conventional buccal	10	150.9996	31.69751	10.02363	128.3245	173.6746	103.75	196.79
	milling buccal	10	14.7773	4.46379	1.41157	11.5841	17.9705	7.36	22.73
	Total	40	44.8847	64.03685	10.12511	24.4047	65.3647	1.52	196.79
OG4	3DPrint buccal	10	7.6778	2.71111	.85733	5.7384	9.6172	4.16	11.88
	cad cam buccal	10	6.4370	2.31455	.73192	4.7813	8.0928	2.64	9.77
	conventional buccal	10	151.2207	29.80947	9.42658	129.8963	172.5451	116.75	198.64
	milling buccal	10	14.6938	3.72872	1.17913	12.0265	17.3612	8.15	19.95
	Total	40	45.0074	63.86092	10.09730	24.5836	65.4311	2.64	198.64

The results of this study, which examined the laboratory data of 40 lithium disilicate crowns fabricated using various conventional, semi-digital, and all-digital methods, are summarized in Table-2 and Table -3. Referring to Tables-2 and -3, and based on the results of the ANOVA test, significant differences were observed among all groups for the examined points, except for the lingual AMD point. In terms of Marginal Gap (MG) buccal, Marginal Gap (MG) lingual, Marginal Discrepancy (AMD) buccal, Axial Gaps (AG2, AG3, OG1, OG3) and Occlusal Gap (OG4), the averages calculated in Table-3 showed significant differences, with lower averages indicating more favorable conditions for the CAD/CAM method. For AG1 and OG2, the averages calculated in Table-3 also indicated significant differences, with lower averages indicating more favorable conditions for the 3D-printing method.

In the case of the AG4 group, the averages calculated in Table-3 showed significant differences, with lower averages indicating more favorable conditions for the Milling method. Although no significant difference was observed in the comparison of lingual AMD among the groups, the CAD/CAM method showed a better average degree of concordance compared to the other groups. Overall, based on this analysis, the conventional group exhibited the weakest performance in terms of adaptation.

## Discussion

The results obtained in this study reject the null hypothesis, indicating that the wax-up method (design) and manufacturing have an effect on the degree of internal and marginal compatibility of lithium disilicate crowns.

In a study conducted in 2016, Shamseddine et al. [34] investigated the marginal and internal compatibility of single lithium disilicate crowns fabricated using conventional and milling methods. The samples were examined using the silicon replica method and scanning electron microscope. To improve the evaluation method and reduce error, the study employed the Micro-CT method. The results showed that the milling group exhibited significantly lower marginal and internal matching compared to the conventional group [34]. However, the findings of the present study contradicted these results, demonstrating that all-digital and semi-digital methods provided better internal and marginal matching compared to the conventional method.

Another study in 2016 by Fathi et al. [35] examined the internal and marginal compatibility of crowns fabricated using milling, conventional wax up, and 3D printing methods. They created 15 wax models using all three methods, and the frames were made during the casting process. The results indicated that the frames obtained from the 3D printer method exhibited higher accuracy in internal and marginal matching compared to the other two methods, with the milling method following closely.

Furthermore, in 2018, Elfar et al. [36] investigated the marginal compatibility of lithium disilicate heat press crowns using three wax pattern making methods: manual or conventional, milling, and 3D printing. The study employed the silicon replica method, digital microscope, and digital image analysis system for quantitative evaluation of adaptation. The Micro-CT method was also utilized to improve evaluation accuracy.

The findings indicated that the 3D printer group exhibited the least marginal gap, significantly outperforming the conventional and milling groups. However, no significant statistical difference was observed between the milling and conventional methods. Importantly, the marginal matching of all restorations fabricated using the aforementioned three methods fell within the clinically acceptable range. On the other hand, in 2018, Homsy et al [37] investigated the internal and marginal compatibility of lithium disilicate inlays made by different methods in a study.

In this study, silicon replica and stereomicroscopy methods were used. In this study, the Micro-CT method was used to improve the evaluation method and reduce the error. Five groups include group one: normal molding and manual wax pattern (CICW group); Group Two: normal molding, laboratory scan of the cast, wax pattern milling (CIDW group); Group Three: normal molding, laboratory scan of the cast, 3D printer of the wax pattern (group CI3DW); Group four: Molding using an intraoral scanner of the chipped tooth, milling a wax pattern (DSDW group); Group five: Molding using an intraoral scanner of the chipped tooth, 3D printer of wax pattern (DS3DW group).

No significant statistical difference was found between CICW, CIDW, CI3DW, and DS3DW groups in marginal maladaptation. They concluded that digitally molded inlays and their wax patterns made using milling have better marginal and internal compatibility than other methods [38]. In the present study, in all the cases where the digital scanning process and wax-up were done digitally, the results obtained were more favorable. The reason for this is the elimination of possible errors and the accumulation of these errors. As in the CAD/CAM method, where the entire method is done digitally, the results were more favorable.

In a 2019 study, Al Hamad et al [38] investigated the internal and marginal compatibility of PFM, lithium disilicate, and zirconia veneers fabricated using different methods. It is important to note that the comparison of different materials in this study may have introduced heterogeneity in the results.

The study used the silicon replica and stereomicroscopy methods, along with the Micro-CT method to enhance evaluation and minimize error. The findings revealed that the all-digital zirconia group exhibited the least axial gap, while the conventional e.max group and conventional/digital PFM group showed the highest axial gap. The conventional e.max group exhibited the least occlusal gap, whereas the digital e.max group showed the highest. The type of crown had no significant effect on marginal, axial, and occlusal alignment, but the construction method significantly influenced axial alignment.

The digital manufacturing process resulted in a significantly smaller axial gap compared to the conventional and conventional/digital methods. The authors concluded that the type of crown and its manufacturing method had no effect on the marginal and occlusal gap of posterior single crowns, but the method of fabrication had a significant effect on the axial gap [36].

In a study conducted by Dolev et al. [39] in 2019, the marginal adaptation of restorations made by heat-press and milling methods was investigated. The study involved designing and making 15 crowns using a CEREC Omnicam intraoral scanner and CEREC MC XL in-office milling machine, as well as IPS e.max CAD blocks. In the second group, 15 crowns were made using the heat press method and IPS e.max press ceramic. The study utilized the silicon replica method and optical microscope to assess marginal adaptation, and the Micro-CT method was employed to improve evaluation accuracy. The average absolute marginal discrepancy was found to be 115 microns in the milling method and 130 microns in the heat press method. Regarding marginal discrepancy, 87 microns were obtained in the milling group and 90 microns in the heat press group.

Based on these data, no significant statistical difference was found between the marginal matching of the two groups. However, in our present study, the CAD/CAM method showed better results in terms of absolute marginal discrepancy and marginal discrepancy compared to the heat-press methods.

In another study conducted by Lim et al. [40] in 2023, the marginal and internal fitness and accuracy of class II inlays fabricated using different methods were investigated. The study compared the conventional method, two milling materials (Lava Ultimate and Zolid Fx multilayer), and 3D printing. The findings showed that the marginal and internal fitness and accuracy of 3D-printed resin inlays and milling of Zolid Fx multilayer were within the clinically acceptable range and were statistically significantly better than other inlays fabricated methods. The authors concluded that 3D-printed resin inlays have a high potential for use in routine clinical practice for esthetic restoration.

In our present study, we found that the all-digital CAD/CAM method exhibited the lowest rate of inconsistency, while the conventional manufacturing method showed the highest rate of inconsistency. In contrast to the aforementioned study, our study revealed that the conventional manufacturing method resulted in the highest axial mismatch. These findings suggest that the construction method significantly influences the degree of internal and marginal adaptation.

It is important to note that various studies conducted in different years have presented varying results regarding the impact of different fabrication methods on the internal and marginal compatibility of lithium disilicate crowns. While our present study suggests that the all-digital CAD/CAM method yields superior results, other studies have reported different levels of effectiveness for various methods. Further research and standardization are necessary to establish consistent findings in this field.

## Conclusion

Considering the limitations of this study, the following can be mentioned:

1. The average matching rate of lithium disilicate crowns fabricated using all-digital and semi-digital methods was better compared to the conventional method. This suggests that digital methods offer improved adaptation and compatibility.

2. The degree of adaptation of lithium disilicate crowns was found to be highest in the CAD/CAM, 3D-print and heat-press wax-up methods, followed by milling and heat-press wax-up, and finally the conventional method. These results indicate that digital fabrication techniques provide superior outcomes in terms of adaptation.

3. Based on the obtained results, it is recommended to use the all-digital method (CAD/CAM) for the treatment of patients requiring lithium disilicate veneers. This approach helps eliminate possible errors and ensures better treatment outcomes.

In light of these findings, it is evident that digital methods, particularly the CAD/CAM approach, offer significant advantages in terms of adaptation and compatibility when fabricating lithium disilicate crowns. Further research and standardization of methods are needed to establish consistent guidelines for optimal fabrication techniques in clinical practice.

## Conflict of Interest

None.
